# Using the readiness assessment for pragmatic trials (RAPT) model to structure partner engagement and strengthen pragmatic research readiness

**DOI:** 10.1017/cts.2026.10726

**Published:** 2026-03-25

**Authors:** Rosa R. Baier, Peter T. Serina, Ann Reddy, Anna Stanislawski, Nate Hunkins, Ellen McCreedy

**Affiliations:** 1 Long-Term Care Quality & Innovation Lab, Brown University School of Public Health, USA; 2 Department of Health Services, Policy & Practice, Brown University School of Public Health, USA; 3 Emergency Medicine, Mass General Brigham Salem Hospital, USA; 4 Bluestone Physician Services, USA

**Keywords:** Embedded pragmatic trials, readiness assessment, partner engagement, translational science, learning health systems

## Abstract

Partner engagement is critical for embedded pragmatic research, yet few structured methods guide early collaboration to strengthen feasibility and mutual understanding. In this case study, Brown University researchers and leaders from a US-based medical practice, Bluestone Physician Services, used the readiness assessment for pragmatic trials (RAPT) model to guide structured discussion and qualitative readiness assessment of a partner-identified intervention concept aimed at improving the timing of palliative care services. Collaborative completion of RAPT created a shared process for assessing feasibility, contextual fit, and alignment with Bluestone priorities. The exercise identified domains needing refinement and guided planning to strengthen data, workflows, and measurement, demonstrating that RAPT can serve as a practical framework for early-stage co-design in pragmatic research.

## Introduction

Embedded pragmatic clinical trials (ePCTs) are studies that evaluate interventions under real-world conditions, testing not only effectiveness but whether they can be implemented and sustained within existing systems [1,2]. These trials depend on effective partnerships between researchers and healthcare providers to ensure that interventions are feasible, acceptable and aligned with health system priorities [1,3] Yet engagement is often informal, without structured approaches to assess context or strengthen alignment before implementation.

The readiness assessment for pragmatic trials (RAPT) model was developed to help research teams evaluate the extent to which an intervention is prepared for pragmatic testing [4]. RAPT includes nine domains spanning evidence, implementation protocol, risk, feasibility, alignment, cost, measurement, acceptability, and potential impact. Although typically used as a research self-assessment, RAPT can also provide a shared framework for researchers and partners to examine feasibility, surface barriers, and identify priorities for refinement before launching a trial.

The US-based methods case study demonstrates how a structured framework can help research teams to identify contextual factors that would otherwise undermine feasibility, and how readiness assessment can evolve into and support active co-design and collaboration. We describe how RAPT was applied collaboratively to engage a physician group during ePCT planning. Using RAPT during early partner engagement may offer several advantages, for example enabling structured discussion of operational context, a factor linked to implementation success in ePCTs [5], and dialog that builds trust and accountability central to learning health system research [6,7].

## Materials and methods

### Setting and partnership context

Bluestone Physician Services provides on-site primary, behavioral, and care-management services for adults in assisted living, memory care, and group home settings across several states in the United States. Leaders from Bluestone and Brown University’s Long-Term Care Quality & Innovation (Q&I) Lab share a commitment to generating evidence that improves care for older adults.

Collaboration between the two groups began in 2016, when we explored research opportunities and Bluestone revised its patient participation agreements to ask patients to consent to the use of their clinical data for research purposes. The partnership first resulted in a project in 2021, when we conducted a researcher-initiated ePCT on advance-care planning (ClinicalTrials.gov NCT04852055), a topic that took on urgency during the early days of the COVID-19 pandemic, when the burden of infection was high in congregate care settings [8] and it was important for assisted living residents being transferred to the hospital to have documented end of life wishes. That ePCT, which predated this case study, established foundational trust and formal processes for our partnership, including regular meetings, shared documentation, and transparent decision-making. These practices aligned expectations and deepened mutual understanding of Bluestone’s workflows, terminology, and priorities, laying the groundwork for subsequent projects [9].

By 2023, Bluestone leaders were initiating their own research ideas and suggested a project to address premature hospice referrals among assisted living residents with dementia, many of whom were later discharged from hospice alive. Bluestone leaders felt that the hospice referrals often reflected unmet behavioral health or palliative symptom needs that Bluestone clinicians could manage internally. They therefore proposed an intervention focused on refining their existing multidisciplinary case review process to better identify patients earlier and align referrals with patients’ needs and goals of care. Bluestone’s existing case review process was largely retrospective, identifying patients only after high-cost hospitalizations. Leaders therefore sought to assess the feasibility of a more proactive approach.

This intervention concept was preliminary, not yet operationalized or protocolized. To ensure that a future trial would be feasible in real-world care delivery, the Brown-Bluestone team used RAPT to structure planning and discussion. The goal was to clarify processes, data needs, and organizational readiness. Because Bluestone leaders were relatively new to formal research partnerships, RAPT also served to explicitly reinforce embedded research requirements and align study design with the day-to-day realities of care delivery.

### The RAPT model

RAPT was developed to help research teams systematically assess whether an intervention and its delivery system are prepared for pragmatic testing [4]. The model includes nine domains: evidence, implementation protocol, risk, feasibility, alignment, cost, measurement, acceptability, and potential impact. Together, these domains reflect the factors most likely to influence successful implementation of an ePCT. Each domain is typically rated along a continuum from low to high readiness and can be summarized graphically to prompt reflection and discussion.

Although designed for self-assessment, RAPT can also structure collaborative reflection with partners, helping identify contextual barriers and priorities for refinement before a trial. In this case study, RAPT served as a facilitation tool to inform early planning for a potential ePCT.

### Application of RAPT

We applied the RAPT model collaboratively to support early-stage planning and qualitative readiness assessment of the proposed intervention. RAPT was used to structure facilitated discussion and reflection about feasibility, context, and alignment across the model’s nine domains. The goal was to use RAPT to focus on which aspects of the proposed approach were sufficiently developed and which required additional refinement prior to pragmatic testing.

The RAPT session occurred in May 2023, three months into a year-long planning project, after meetings to clarify the clinical problem, proposed approach, and organizational context. Prior to the session, participants had access to a written description of the proposed case review process, summary information on Bluestone’s existing workflows, and preliminary discussion of potential outcomes and data sources; no finalized protocol or cost-benefit analysis had been developed. During a recorded Zoom session, participants anonymously rated each domain, reviewed aggregated results in real time, and discussed their rationale. A Brown project director facilitated discussion but did not participate in voting.

Participants rated each domain on a five-point scale anchored at low (1), moderate (3), and high readiness (5) readiness for pragmatic research, based on their individual perspective (Table 1). Ratings were entered anonymously using Zoom polling and displayed in real time, ensuring equal opportunity for input across roles and supporting open discussion. After the anonymous polling captured individual ratings, discussion was then facilitated to support shared understanding of those ratings, jointly consider factors related to feasibility, workflow fit, and data availability, and identify which domains would require additional preparatory work or clarification. Differences in perspective were addressed through discussion, rather than formal adjudication.

**Table 1.** RAPT domain-level polling response choices

Domain & poll prompt	Anchors on 5-point readiness scale		
	1 – Low, Not Ready	3 – Medium, Partially Ready	5 – High, Fully Ready
**Alignment**: To what extent does the intervention align with external stakeholders’ priorities?	*Stakeholders (policymakers, payors, advocates, and others) do not believe the intervention addresses a current or anticipated priority.*	*Some stakeholders believe the intervention addresses a priority.*	*Most or all stakeholders believe the intervention addresses a priority.*
**Implementation Protocol**: Is the intervention protocol sufficiently detailed to be replicated?	*There is no protocol.*	*The protocol provides some documentation but may be difficult to replicate.*	*The protocol is well documented and is likely to be replicable.*
**Acceptability**: How willing are providers likely to be to adopt the intervention?	*Acceptability is unknown or staff are unlikely to believe the intervention is feasible or needed.*	*Acceptability is unknown, but staff are unlikely to believe the intervention is feasible or needed.*	*Acceptability is known and staff believe the intervention is feasible and needed.*
**Evidence**: To what extent does the evidence base support the intervention’s efficacy?	*There are no efficacy studies, or the efficacy studies did not use rigorous methods (e.g., a randomized, controlled trial).*	*A single study using rigorous methods demonstrated efficacy.*	*Multiple studies using rigorous methods have demonstrated efficacy.*
**Measurement**: To what extent can outcomes be captured during the conduct of the pragmatic trial?	*Outcomes cannot be captured without major modifications to systems (e.g., clinical assessments, documentation, or electronic health records) or increases in staff time.*	*Outcomes can be captured with minor modifications to systems or increases in staff time.*	*Outcomes are already routinely captured.*
**Risk**: Is it known how safe the intervention is?	*The risks (harms and discomforts) are unknown or are known to be more than minimal (e.g., greater than ordinarily encountered in daily life).*	*The risks are unknown but are likely minimal.*	*The risks are known to be minimal.*
**Feasibility**: To what extent can the intervention be implemented under existing conditions?	*Resources necessary for implementation (e.g., staff, infrastructure, payment) are absent or insufficient.*	*Minor modifications to existing resources would enable implementation.*	*Implementation is possible with existing resources.*
**Cost**: Is it known how safe the intervention is?	*Cost–benefit/cost-effectiveness analysis has not been completed (formally or informally) and it is unknown whether benefits outweigh costs.*	*Cost–benefit/cost-effectiveness analysis has not been completed, but benefits are likely to outweigh costs.*	*Cost–benefit/cost-effectiveness analysis demonstrates benefits outweigh costs.*
**Impact**: How useful will the results be?	*Providers and stakeholders (policymakers, payors, advocates, and others) are unlikely to believe that the outcomes are useful (e.g., to inform clinical care or policy).*	*Some providers or stakeholders are likely to believe the outcomes are useful.*	*Most or all providers and stakeholders are likely to believe the outcomes are useful.*

RAPT = readiness assessment for pragmatic trials.

### Data sources and analysis

The RAPT session generated domain ratings, participant comments, and facilitator notes. Following the session, the research team summarized domain ratings, rationale, and illustrative quotes, then shared this summary with Bluestone partners for their review and agreement regarding interpretation and next steps. Analysis focused on domains of low or moderate readiness and related contextual factors. Findings are summarized in Table 1 to highlight participants’ reasoning and contextual insights. All materials are available on request.

### Ethical considerations

This project focused on providers’ professional experiences and organizational processes to inform future research design and was not considered human subjects research; institutional review board review was not required.

## Results

The May 2023 discussion included eight participants: five Bluestone leaders (members of the executive team, a data analyst, and a physician) and three Brown researchers (two faculty investigators, including a physician, and a data analyst). A Brown project director facilitated. The group assessed a proposed intervention: a risk-score algorithm designed to flag patients at high six-month mortality risk and trigger provider-led end-of-life care plan reviews, advance care planning conversations, and related care coordination actions.

The RAPT assessment and discussion clarified data and workflow needs and identified areas requiring further definition before an ePCT. Overall qualitative readiness levels reflect collective understanding following discussion, which focused on understanding the rationale for differing perspectives rather than forcing agreement or consensus.

Table 2 summarizes domain-level assessments and rationale. Participants judged readiness low in two domains (*Alignment* and *Implementation Protocol*), moderate in three (*Acceptability*, *Evidence*, and *Measurement*), and high in four (*Risk*, *Feasibility*, *Cost*, and *Impact*). These assessments reflected Bluestone’s stage of operational planning: the proposed approach was conceptually aligned with organizational priorities, but workflow, documentation, and data linkage required further development.

**Table 2.** RAPT domain-level readiness assessments with selected illustrative partner quotes

RAPT domain	Readiness	Rationale/selected illustrative quote
**Alignment**	Low	Variation in hospice and assisted living policies would require cross-organization coordination. *“Right now hospice providers provide a bunch of free resources to assisted living facilities. I think a lot of that can be accounted for in process and I do think it’s overcomeable.”*
**Implementation protocol**	Low	Needed clearer definition of patient eligibility, team roles, and documentation procedures. *“With protocol refinement [we need] specificity on who we are referring to, what type of care management … and people will be able to sink their teeth into it a little bit more.”*
**Acceptability**	Moderate	Partner clinicians were supportive, but noted potential workflow burden. *“I’m very optimistic about this … I just think this is going to create some structure for them that is desperately needed.”*
**Evidence**	Moderate	Limited prior evidence specific to assisted living. *“It’s trying to find something very specific to assisted living patients. That’s difficult for sure.”*
**Measurement**	Moderate	EMR and claims data existed, but were not linked. *“We need specificity on the actual outcome measures … connecting the dots between data sources, that’s the challenge.”*
**Risk**	High	Minimal patient or organizational risk anticipated. *“I did a quick risk-mitigation thought processing here, and most of the interventions don’t have a deleterious component to them.”*
**Feasibility**	High	Built on existing case-review processes. *“I think it’s work that’s already done [by providers]. It’s just putting some different parameters around it.”*
**Cost**	High	Expected to be cost-neutral within current operations. *“[I can’t see] why it wouldn’t be economically viable.”*
**Impact**	High	Believed to improve patient-centeredness and continuity of care. *“Any intervention where we’re spending more time with the patient … that’s impactful because you’re provisioning care and making an effort to provision better care.”*

EMR = electronic medical record; RAPT = readiness assessment for pragmatic trials.

The *Alignment* domain, which focuses on fit with external stakeholders’ priorities, policies, and incentives, was assessed as low because the team recognized that improving the timing of hospice referrals would require coordination across multiple organizations, including hospice providers and assisted living communities whose policies and incentives varied widely. The *Implementation Protocol* domain was also assessed as low, reflecting the need to specify which Bluestone patients would be targeted, which team members would lead case reviews, and how recommendations would be documented and followed.

Domains assessed as moderate revealed additional considerations for planning. *Acceptability,* which focuses on appropriateness and buy-in within the partnering health system, reflected enthusiasm among Bluestone clinicians tempered by recognition that new workflows could create initial burden. *Evidence* was assessed as moderate because the team identified few evidence-based models directly applicable to assisted living residents. *Measurement* was also assessed as moderate, reflecting uncertainty about how best to capture outcomes pragmatically using existing electronic health records and claims data.

Domains assessed as high (*Risk, Feasibility, Cost,* and *Impact*) reflected the team’s belief that the proposed approach posed minimal patient or organizational risk, could be implemented within existing structures, and aligned with Bluestone’s organizational priorities around proactive, patient-centered care.

Overall, the RAPT session helped partners articulate key data, workflow, and organizational factors relevant to the proposed pragmatic study. Participants identified gaps in readiness related to patient eligibility definitions, delineation of team roles within the case review process, and the need for reliable linkages between electronic health record and claims data to support outcome measurement. The discussion surfaced workflow dependencies and operational constraints that had not been fully articulated during earlier planning conversations, including questions of workflow ownership, outcome definitions, and data availability. In addition, participants highlighted variability in external hospice and assisted living practices that would require coordination beyond Bluestone’s direct control as a physician practice.

Together, these findings informed identification of priority areas requiring further development before advancing to pragmatic testing. As a result of the RAPT discussion, the team identified the need to strengthen data infrastructure, clarify measurement strategies, refine workflows, and expand participation to include additional Bluestone leaders, such as the Senior Director of Value-Based Care Programs. Subsequent planning activities included examining claims data to identify patterns of hospice live discharges, reviewing electronic health record data sources to support outcome measurement, and assessing evidence-based practices relevant to assisted living populations. The discussion also prompted development of a mortality risk score to support proactive identification of patients most likely to benefit from goal-based, prognosis-driven interventions and appropriate timing of hospice enrollment.

## Discussion

This methods case study examined how the RAPT model was applied to structure partner engagement during early trial planning. Using RAPT in partnership with Bluestone Physician Services leaders demonstrated how a readiness framework can also serve as a facilitation tool for partner engagement and co-design. Beyond generating qualitative assessments, the process structured reflection, promoted transparency, and supported shared ownership of research decisions. Grounded in anonymous polling, collective interpretation, and equal opportunity for input, the discussion format created a balanced environment for researchers and provider partners to explore feasibility, contextual fit, and workflow alignment before developing and implementing an ePCT protocol. This approach aligns with learning health systems and partnered research principles that emphasize mutual trust, accountability, and knowledge generation [6,7].

These findings underscore the value of using readiness assessment as an early translational strategy rather than solely as a pre-trial checkpoint. In this case, applying RAPT during early planning revealed gaps related to intervention definition, workflow ownership, data integration, and external coordination that could have limited feasibility had a trial proceeded prematurely. Identifying these issues prior to protocol development allowed the partnership to sequence preparatory activities deliberately, strengthening operational alignment and shared understanding before advancing toward pragmatic testing. This use of RAPT illustrates how readiness frameworks can support early co-design and improve the translational trajectory of partner-embedded research.

This experience highlights two complementary functions of readiness assessment. Conceptually, RAPT evaluates whether an intervention and delivery system are ready for pragmatic testing [4] In practice, applying it collaboratively shifts the tool from a diagnostic checklist to a participatory planning framework. The exercise encouraged reflection on both operational and relational readiness, aligning expectations and surfacing contextual barriers that might otherwise have undermined implementation feasibility and fidelity in a trial.

The discussion highlighted the importance of the *Alignment* and *Implementation Protocol* domains, which focus on an intervention’s contextual fit with organizational priorities and its replicability within complex systems. These domains encourage researchers to engage around priorities, incentives, and workflow realities, developing a more nuanced understanding of context and recognizing providers as experts in their environments and co-leaders in the research process. By identifying issues early, the Brown-Bluestone team could focus subsequent planning on operational and data considerations rather than troubleshooting barriers once a study was underway. Other teams may find similar value in using RAPT to structure early dialog about feasibility and contextual fit during proposal development or pilot design.

This case study has several limitations. It reflects the experience of a single research-provider partnership and one facilitated RAPT session during early planning, which may limit generalizability to other contexts and stages of pragmatic trial development. However, several practical insights emerged. The use of anonymous polling, joint review of results, and documentation of participants’ reasoning enhanced transparency and facilitated shared learning. For research teams seeking to build sustainable partnerships, RAPT offers a low-burden, adaptable structure for group discussion and reflection that can strengthen both the quality and feasibility of embedded pragmatic research.

Since the completion of this project, RAPT-Indigenous (RAPT-I) has expanded the RAPT framework to explicity incorporate domains focused on context, equity, partnership, scalability, and sustainability [9] Although developed for research with Indigenous populations, these domains reflect considerations broadly relevant to pragmatic research. Their inclusion reinfoces the importance of shared leadership, contextual fit, and sustainability from the outset of study planning. Future research could explore how RAPT and RAPT-I can be used to engage partners and support more equitable and contextually grounded pragmatic research.

## Conclusion

Applying RAPT in partnership created a structured space for dialog between researchers and provider partners, consistent with principles of partner-engaged research [7]. The process helped establish shared understanding of feasibility and contextual fit, clarified workflow and data needs, and identified domains requiring additional attention before launching a trial. More broadly, this case demonstrates how a readiness framework can be repurposed as a practical method for partner engagement, offering a structured and replicable approach to co-designing feasible, contextually grounded pragmatic research.
